# Infection control and radiation safety practices in the radiology department during the COVID-19 outbreak

**DOI:** 10.1371/journal.pone.0279607

**Published:** 2022-12-27

**Authors:** Mohamed M. Abuzaid, Wiam Elshami, H. O. Tekin

**Affiliations:** 1 Medical Diagnostic Imaging Department, College of Health Sciences, University of Sharjah, Sharjah, UAE; 2 Istinye University, Faculty of Engineering and Natural Sciences, Computer Engineering Department, Istanbul, Turkey; Universiti Teknologi Malaysia - Main Campus Skudai: Universiti Teknologi Malaysia, MALAYSIA

## Abstract

**Rationale and objectives:**

Radiology personnel must have good knowledge, experience and adherence to radiation protection and infection control practices to ensure patient safety and prevent the further spread of the COVID-19 virus. This study analysed compliance and adherence to radiation protection and infection control during COVID-19 mobile radiography.

**Methods:**

A cross-sectional using online survey was conducted from September to December 2021. Data on demographic characteristics, adherence to radiation protection and infection control practice were collected during mobile radiography for COVID-19 patients in the study. A random sample of the radiographers working in COVID-19 centres in the United Arab Emirates.

**Results:**

Responses were received from 140 participants, with a response rate of 87.5%. Females were the predominant participants (n = 81; 58%). Participants aged ages between 18–25 years (n = 46; 33%) and 26–35 years (n = 42; 30%), (n = 57; 41%) had less than five years of experience, followed by participants who had more than 15 years (n = 38; 27%). Most participants (n = 81; 57.9%) stated that they performed approximately 1–5 suspected or confirmed COVID-19 cases daily. The participants had moderate to high adherence to radiation protection, with a mean and standard deviation of 42.3 ± 6.28. Additionally, infection control adherence was high, with 82% of the participants showing high adherence.

**Conclusion:**

Continuous guidance, training and follow-up are recommended to increase adherence and compliance to radiation protection and infection control compliance. Educational institutions and professional organisations must collaborate to provide structured training programmes for radiology practitioners to overcome the practice and knowledge gap.

## Introduction

Radiology personnel are at the frontline of the fight against COVID-19. Still, most of them have little experience working with COVID-19 patients [1]. Additionally, nonstandard preventative techniques used during radiological investigations increase the danger of hospital cross-infection in the radiology department [2].

The (COVID-19).-19 outbreak was reported in Wuhan, Hubei province, in late December 2019, quickly spreading throughout China and worldwide by March 2020 [3]. Radiology professionals are near in touch with suspected and confirmed patients and are at significant risk of occupational exposure to the disease. Previous studies have shown that front-liners account for about 10–20% of all COVID-19 cases [4]. The risk of positive COVID-19 tests was 3–4 times higher among healthcare workers compared to the general community [5]. The spread of COVID-19 among healthcare workers contributed to further spread in the community, directly affecting healthcare services.

Radiological examinations play a critical role in diagnosing and managing COVID-19 cases [1, 6–8]. Chest X-ray and computed tomography (CT) examinations are the most effective imaging procedures for COVID-19 screening and diagnosis. Standard infection prevention techniques should be followed strictly to prevent hospital cross-infection from radiological studies. Detailed infection control measures were prepared internationally for radiology professionals for the disinfection of equipment, auxiliary instruments, and the workplace to reduce infection risk among workers, visitors and patients [2, 9].

Mobile chest X-ray (CXR) is performed as a daily routine procedure for COVID-19 patients admitted to intensive care units. During radiology examinations, radiology professionals must provide radiation protection to patients. During mobile examinations, implement workers must perform the ‘as low as reasonably achievable’ (ALARA) procedures by employing the shortest possible exposure time, ensuring the optimum distance between the radiation source and the patient and applying proper patient, self and staff shielding [10, 11]. The study was distinctive since it was the first to examine how radiation protection and infection control were practiced during the COVID-19 pandemic in the radiology department. The sudden outbreak of COVID-19 required quick recommendations about the roles and responsibilities of healthcare practitioners involved in managing COVI-19 cases. Radiographers are specifically trained to use imaging to benefit patients; they are aware of potential risks from ionising radiation and need to minimise the harm from inappropriate or excessive radiation use. In addition, other aspects of radiographer practice encompass elements of infection control. In this paper, we attempt to investigate the adherence to patient safety that forms part of normal practice for radiographers during the COVID-19 pandemic.

## Methods

### Study design and setting

A descriptive cross-sectional study using an online survey was conducted among radiographers working in in hospitals and health centres affiliated with COVID-19 cases in the United Arab Emirates (UAE). Radiographers who practice in the “Northern Emirates,” the five emirates located in the North of the country, were invited to participate in the study. The data collection duration was four months (August to December), with regular reminders to maximize response. This study followed the Strengthening the Re- porting of Observational Studies in Epidemiology (STROBE) reporting guideline.

### Participants

Radiographers who worked in the hospitals and clinics under the Ministry of Health and Prevention (MOHAP) authority and were willing to participate during the study period provided the data. Radiographers who failed to complete the survey by the deadline were not included.

### Sample size

Random sample based on MOHAP records, there were 272 radiographers worked in different location [11]. A sample size of 140 was determined to have appropriate power based on a confidence level of 95% and margin of error of 5%.

### Instrument for data collection

A self-administrated questionnaire was designed in English language, reviewed and piloted by a group of five senior radiographers and three infection control officers. The survey’s readability and appropriateness, as well as the questions’ definition, comprehension, and consistency were all tested during the pilot. The information statements and permission forms was also evaluated [11]. Additionally, the reviewer responses were used to improve the order and layout of the questions to reduce dropout rates. A pilot study was conducted on 15 systematic randomly selected participants whose results were excluded from the main study. A pilot study was conducted to ensure the understandability of the questionnaire. Internal consistency reliability of the questionnaire conducted using Cronbach’s Alpha.

### Survey

The first part of the questionnaire consisted of demographic characteristics (e.g. age, academic qualification, work experience, the average number of COVID-19 cases performed daily and any special training or allowance during the pandemic). In the second part, it was investigated whether the radiation protection practices of participants minimise radiation exposure for workers staff and patients. Compliance with the following measures was assessed; wearing thermoluminescence dosimeter (TLD), lead aprons, thyroid collars, collimation, distance shielding, gonad shielding and proper exposure parameter. Finally, compliance with infection control practices was evaluated in the third part: personal protective equipment (PPE), infection precaution, equipment disinfection, hand hygiene and following the standard documentation protocol.

A 4-point Likert scale was used in the survey with the following scores: (4) always, (3) often, (2) sometimes and (1) never. The higher the score, the better the practice. The score was transformed into a percentage scale by dividing the total score by the maximum possible score multiplied by 100. Accordingly, the score was categorised as poor adherence (<60%), moderate adherence; (60–80%) and good adherence: (>80%) (11). A research assistant handed out hard copies of the survey to 160 radiographers, information papers, consent forms and explanations of the objectives of the study.

### Ethical considerations

The research was approved by the Institutional Research Unit in University of Sharjah. The objectives of the study were explained and participants’ privacy was guaranteed. The participants were informed that they were free to withdraw at any time during the data collection process. Written informed consent was also obtained from all respondents prior to their involvement in the study, using an online consent form in google form, those who provided consent then completed a secure online survey.

### Statistical methods

Data were collected, and graphs were created with Microsoft Office Excel 2016 (Microsoft Corporation CA, USA) and analysed in the Statistical Package for Social Sciences (SPSS) IBM SPSS Statistics for Windows, Version 25.0. Armonk, NY: IBM. The descriptive statistics for the questions in all four sections were completed. ANOVA was conducted to understand the differences between radiation protection and infection control practices based on demographic differences. Jarque-Bera test of normality of distribution was conducted and the level of statistical significance used is α = 0.05.

## Results

### Demographics and background of participants

Responses were received from 140 participants, making the response rate 87.5%, predominantly females (58%). Most of the participants were aged between 18–25 years (33%). Most respondents had a bachelor’s degree (77%) and Most had less than five years of experience (41%). Around (56%) of the respondents stated that they received special training related to COVID-19, while (24%) stated they received special allowances/incentives during the COVID-19 pandemic “Table 1”.

**Table 1:** Demographic data of the participants of the study.

		Frequency (%)
Gender	Male	59 (42)
	Female	81 (58)
Age (years)	18–25	46 (33)
	26–35	42 (30)
	36–45	29 (21)
	46–65	23 (16)
Qualification	Diploma	10 (7)
	Bachelor’s	108 (77)
	Master’s	22 (16)
Experience	1–5 years	57 (41)
	6–10 years	21 (15)
	11–15 years	24 (17)
	More than 15 years	38 (27)
Number of COVID-19 cases handled per day	1–5	81 (58)
	6–10	48 (34)
	11–15	11 (8)
COVID-19 related training	Yes	78 (56)
	No	62 (44)
Allowances/incentives received	Yes	33 (24)
	No	107 (76)

### Radiation protection practice during COVID-19 mobile cases

This section of the questionnaire had 10 sub-questions on a 4-point Likert scale. Responses were scored 1–4 as (1) never, (2) sometimes, (3) most of the time and (4) always. The frequencies of the responses and the distribution are given in “Tables 2 and 3”. When the participants were asked if they wore TLD, most (61.4%) stated they always wore it. Less than half (45%) of the respondents stated they always wore a lead apron and most (55%) participants never wear thyroid collars when performing mobile imaging procedures. Many respondents stated that they practised proper collimation always (46%) and most of the time (37%). Most participants (44%) responded ’always’ for using proper SID/FFD during imaging. Around (36%) of the participants responded ‘sometimes’ when asked if they applied patient gonad shielding and 30% (n = 42) for applying patient lead shielding during procedures. The minimum exposure time was applied most of the time, as stated by most participants (43%). A total of (37%) of participants stated that they use the lead apron for all co-patients or staff most of the time. Closing the door was a predominant practice, as most participants (57%) stated that they always performed this procedure.

**Table 2:** Radiation protection practice frequencies are represented as total amount of respondents and the respective percentage.

	Never	Sometimes	Most of the time	Always
1. Wearing TLD	21(15)	15(11)	18(13)	86(61)
2. Wearing lead apron	16(12)	39(28)	22(16)	63(44)
3. Wearing thyroid collar	77(55)	32(23)	16(12)	15(11)
4. Using proper collimation	1(1)	22(16)	52(37)	65(46)
5. Using proper SID/FFD	2(2)	29(21)	47(33)	62(44)
6. Apply patient gonad shielding	18(13)	50(36)	34(24)	38(27)
7. Apply patient lead shielding	16(12)	42(30)	40(29)	42(30)
8. Using minimum exposure time	2(2)	18(13)	61(43)	59(42)
9. Using the lead apron for all co-patient/staff	10(7)	35(25)	52(37)	43(31)
10. Closing the room door	1(1)	8(6)	50(36)	81(57)

**Table 3:** Infection control practice frequencies are represented as total amount of respondents and the respective percentage.

	Never	Sometimes	Most of the time	Always
1. Wearing personal protective gear, facemask, gloves, face shield, etc.	3(2)	20(14)	20(14)	97(70)
2. Appropriate isolation precaution practices maintained during mobile radiography	5(4)	20(14)	32(23)	83(59)
3. Equipment disinfected according to Infection Control policies and procedures	5(4)	17(12)	38(27)	80(57)
4. Hand hygiene (personal cleanliness)	2(1)	13(9)	33(24)	92(66)
5. Standardised hospital protocols for decontaminating imaging equipment after the imaging procedure	5(4)	11(7)	32(23)	92(66)

### Infection control practice during COVID-19 mobile cases

This section of the questionnaire had five sub-questions in which responses were recorded on a 4-point Likert scale, which were scored as never (1), sometimes (2), most of the time (3), and always (4). The majority of the respondents (70%) stated they always wore personal protective gear, such as a facemask, gloves and face shield. Nearly (59%) of the participants “always” used appropriate isolation precaution practices, and (57%) “always” disinfected the equipment according to infection control policies and procedures. Regarding personal hygiene, around (65.7%) of the participants always maintained hand hygiene and (66%) always followed standardised hospital protocols for decontaminating imaging equipment after the imaging procedure.

### Knowledge and special training

Most of the respondents (71%) stated that they were up-to-date and aware of the latest information regarding COVID-19. Around (69%) of them attended the COVID-19 awareness course. Additionally, half of the respondents (50%) stated that they obtained support from the radiology department.

The participants were asked to what extent they have confidence in handling suspected COVID-19 patients and most participants (49%) stated they were confident to a great extent. Additionally, most respondents (66%) indicated that health organisations were the primary sources of information about COVID-19.

### Comparison of demographics and responses

The demographics of the participants and their responses were analysed through ANOVA. The responses of radiation protection practices and infection control practices were summed, with the lowest possible score of 15, which meant that the worker did not adhere to best practices and the highest possible score of 60, which meant that the worker followed best practices.

A significant association was found between the educational qualifications of the respondents and their best practices (p = 0.0009, α = 0.05). The mean score of the respondents with Master degree (μ = 43.5) and Bachelor degree (μ = 48.1) were higher than those with a diploma (μ = 40.7). There was no significant association revealed among the radiographers’ best practises and the training provided (p = 0.10, α = 0.05), age (p = 0.34, α = 0.05), gender (p = 0.59, α = 0.05), number of COVID-19 cases they handled (p = 0.35, α = 0.05), and their experiences (p = 0.35, α = 0.05).

### Adherence to radiation protection and infection control

The adherence of the participants to radiation protection and infection control is shown in “Table 4”. The lowest score in the study was 21, and the maximum score was 57 in terms of radiation protection practices. The mean and standard deviation (SD) was 42.3 ± 6.28. The study found a minimum score of seven and a maximum score of 20 in infection control practices, with a mean and SD of 17.3 ± 3.49. The percentage of participants who adhered to good radiation protection was 50%, while that of those who adhered to infection control was 82%.

**Table 4:** Scoring of the adherence to radiation and infection control practice.

		(n = 136)	
Radiation Protection Practice during COVID-19 Mobile Cases	Min-Max, Mean ± SD	21–57	42.3 ± 6.28
	Poor adherence (N/%)	27	19%
	Moderate adherence (N/%)	43	31%
	Good adherence (N/%)	70	50%
Infection Control Practice during COVID-19 Mobile Cases	Min-Max, Mean ± SD	7–20	17.3 ± 3.49
	Poor adherence (N/%)	14	10%
	Moderate adherence (N/%)	11	8%
	Good adherence (N/%)	115	82%

Poor adherence: ≤60%; Moderate adherence: 60–80%, and Good adherence: ≥ 80%.

## Discussion

During the COVID-19 pandemic, there was a need for rapid and accurate diagnostic procedures. The importance of medical imaging (chest radiography and computed tomography) in the fight against COVID-19 has been confirmed [6, 8]. Patients, professionals and public safety are crucial during medical imaging investigations. Medical imaging professionals must gain knowledge and abilities to ensure patient safety and up-to-date information while the pandemic continues. Various studies and papers have been published emphasising patient safety in medical imaging during COVID-19 [2, 9, 12–14] and the challenges and optimisation strategies in radiology service during the COVID-19 pandemic [15] but no studies investigated the practice during mobile radiography. Increased utilisation of mobile radiography during the COVID-19 pandemic required more attention to occupational and patient doses [1]. Therefore, in April 2020, the International Society of Radiographer and Radiologic Technologist (ISRRT) released a response paper to ensure patient safety and radiation protection during medical imaging procedures during COVID-19 cases [9].

During mobile radiography, the radiation safety goals were determined not to exceed occupational exposure limits for workers or the general public, perform optimisations, use protective garments, monitor occupational exposure, request justification, correct patient identification and reduce repeats. Established radiation protection rules and practices must be followed by radiology departments to ensure the safety of workers, patients and the environment.

It was revealed in the results that most participants (81%) had high to moderate adherence to radiation protection practices during mobile radiography. Regarding personal safety, the findings are both surprising and alarming, as radiology professionals should be aware of the importance of wearing TLDs, lead aprons and thyroid collars during their practice. The participants who never wore TLD (15%) or lead apron (12%) showed an indication of negligence or a low awareness level. Wearing TLDs is critical for personal safety and is used to alert workers and hospitals when radiation levels exceed acceptable limits to avoid the danger of radiation exposure [16]. Additionally, the lead apron is a critical element of personal radiation protection [17].

The results of wearing the thyroid collar are linked to its unavailability and a lack of knowledge about the importance of wearing it during mobile radiography. These results are similar to those obtained in other studies [11–15]. Therefore, additional specialised radiation safety training is required, focusing on the risk of radiation exposure in the workplace and the significance of wearing TLDs while working.

Regarding patient protection, moderate to high adherence concerning collimation, distance, exposure time and gonad shield was shown in the results “Table 2”. A fundamental concept to reduce radiation exposure is (ALARA). According to ALARA, minimum exposure time, distance to radiation and radiation shielding are the three key parameters to decrease occupational exposure to scatter radiation.

Concerning infection control, PPE is based on the risk of exposure by the activity being performed and the transmission dynamic of the virus considering the three types of transmission of diseases: contact, droplet and aerosol. Therefore, appropriate PPE for droplets is mandatory to reduce the spread of COVID-19, including eye protection, either goggles or a face shield and a facemask, depending on the procedures being performed [14–18]. During mobile radiography, infection prevention measures include the imaging room and equipment. This must be regular cleaning and decontamination [19].

High to moderate adherence to infection control practices was shown in this study. The participants indicated good adherence to PPE during mobile radiography, with 70% always answering and 14% answering most of the time. However, no total adherence of 100% during COVID-19 cases has been shown in other studies [20].

The percentage of 2% of staff workers who indicated never using PPE could be justified either by poor practice or PPE shortage. Hand hygiene adherence was up to the international level and accepted, with 66% of the workers answering ‘always’, 24% answering ‘most of the time’, 9% answering ‘sometimes’ and only 1% stating ‘never’. Better hand hygiene adherence and performance compared to PPE adherence could be related to knowledge and risk awareness.

Only 50% of those polled said they obtained support from the radiology department, and only 24% said they received incentives throughout the pandemic. The payment system for healthcare providers was adjusted by many healthcare systems worldwide during the pandemic [21]. Personnel motivation and support are processes with significant economic and societal implications.

### Limitation

This work focused on radiographers’ practice during mobile radiography. We acknowledge some limitations. First, we did not collect full details of radiation and occupational dose during the COVID-19 period. Second, we investigated only the radiographer’s practice, whereas many healthcare professionals such as nurses and radiologists are involved in mobile radiography. In future studies, we plan to target a larger population, other professionals and collect qualitative data.

## Conclusion

The delivery of medical imaging services faces tremendous challenges in an exceptional pandemic. The delivery of healthcare is continuing despite the crisis, and given the crucial role that medical imaging services play in the ongoing battle against COVID-19, the standard and safety of care are becoming increasingly crucial. The current study highlights the current level of radiographer adherence to patient safety during COVID-19, which still has room for improvement to meet its goal of complete COVID-19 eradication. Mobile radiography examinations have an essential role in diagnosing and following COVID-19 patients. Therefore, radiology personnel must have good knowledge, experience and adherence to radiation protection and infection control practices to ensure patient safety and prevent the further spread of the virus. Based on ALARA and infection control practices, such as hand hygiene and PPE, proper radiation protection measures are among the most critical infection control and prevention measures. Continuous guidance, training and follow-up are recommended to ensure increased compliance and, as a result, a decreased rate of cross-infection and probable fatalities. The health organization should invest in a variety of COVID-19 prevention initiatives, such as health education, increase community awareness, the implementation of artificial intelligence applications, and improvements to its preventative procedures, in order to achieve the highest level of patient safety during mobile radiography.

## Supporting information

S1 Data (XLSX)
